# Phasic excitation of ventral tegmental dopamine neurons potentiates the initiation of conditioned approach behavior: parametric and reinforcement-schedule analyses

**DOI:** 10.3389/fnbeh.2014.00155

**Published:** 2014-05-06

**Authors:** Anton Ilango, Andrew J. Kesner, Carl J. Broker, Dong V. Wang, Satoshi Ikemoto

**Affiliations:** Behavioral Neuroscience Branch, National Institute on Drug Abuse, National Institutes of HealthBaltimore, MD, USA

**Keywords:** phasic firing, optogenetics, conditioning, operant, reinforcement schedule, approach motivation, reward

## Abstract

Midbrain dopamine neurons are implicated in motivation and learning. However, it is unclear how phasic excitation of dopamine neurons, which is implicated in learning, is involved in motivation. Here we used a self-stimulation procedure to examine how mice seek for optogenetically-induced phasic excitation of dopamine neurons, with an emphasis on the temporal dimension. TH-Cre transgenic mice received adeno-associated viral vectors encoding channelrhodopsin-2 into the ventral tegmental area, resulting in selective expression of the opsin in dopamine neurons. These mice were trained to press on a lever for photo-pulse trains that phasically excited dopamine neurons. They learned to self-stimulate in a fast, constant manner, and rapidly reduced pressing during extinction. We first determined effective parameters of photo-pulse trains in self-stimulation. Lever-press rates changed as a function of the manipulation of pulse number, duration, intensity, and frequency. We then examined effects of interval and ratio schedules of reinforcement on photo-pulse train reinforcement, which was contrasted with food reinforcement. Reinforcement with food inhibited lever pressing for a few seconds, after which pressing was robustly regulated in a goal-directed manner. In contrast, phasic excitation of dopamine neurons robustly potentiated the initiation of lever pressing; however, this effect did not last more than 1 s and quickly diminished. Indeed, response rates markedly decreased when lever pressing was reinforced with inter-reinforcement interval schedules of 3 or 10 s or ratio schedules requiring multiple responses per reinforcement. Thus, phasic excitation of dopamine neurons briefly potentiates the initiation of approach behavior with apparent lack of long-term motivational regulation.

## Introduction

Midbrain dopamine (DA) neurons play important roles in both learning, which guides behavior, and motivation, which invigorates it (Ikemoto, 2007). DA neurons fire in two notable ways: low-frequency tonic firing and high-frequency phasic firing. Electrophysiological studies have found that the latter occurs upon unexpected presentation of salient stimuli (Schultz et al., 1997; Bromberg-Martin et al., 2010), thus providing incidental information about the external environment. This type of phasic firing is characterized as a “reward prediction errors” (Montague et al., 1996; Schultz et al., 1997) and contributes to associative (or trial-and-error) learning (Steinberg et al., 2013), to maximize the procurement of rewards from the environment.

In addition to participating in associative learning, phasic excitation of DA neurons may have positive motivational effects. Pharmacological studies established dopamine's role in motivation and reward (Wise and Rompre, 1989; Ikemoto and Panksepp, 1999; Ikemoto, 2007); in particular, pharmacological agents that increase DAergic signaling are readily self-administered into the ventral striatum (Ikemoto et al., 1997, 2005; Ikemoto, 2003; Shin et al., 2008), a major projection area of DA neurons localized in the ventral tegmental area (VTA). DA receptor antagonist injections into the ventral striatum appear to disrupt the initiation of, but not ongoing, approach responses (Nicola, 2010). However, such studies employed pharmacological manipulations that modulate DA systems on the order of minutes or longer; therefore, it is unclear how phasic signals of DA neurons, which typically last no more than a half second at a time, play a role in motivation and reward. Optogenetic procedures allow experimenters to selectively stimulate DA neurons in phasic-firing manners. A recent optogenetic study began to shed light on this issue and found that phasic, but not tonic, stimulation of DA neurons induces conditioned place preference (Tsai et al., 2009).

The first aim of the present study was to determine the most effective parameters of photo-pulse trains that phasically excite VTA DA neurons as reinforcer. Specifically, we manipulated the duration, intensity, frequency, and number of pulses of photo-pulse trains stimulating the opsin channelrhodopsin-2 (ChR2) expressed on VTA DA neurons, and examined their effects on lever-press rates in an operant reinforcement procedure, often referred to as “self-stimulation.” Such information has not yet been systematically examined and is important in designing and interpreting behavioral studies on functions of phasic firing of DA neurons.

Phasic excitation of VTA DA neurons appears to be a powerful reinforcer. We and others have observed that phasic stimulation of VTA DA neurons is so rewarding that animals engage in fast, constant self-stimulation behavior while keeping any other activity minimal, resulting in hundreds of presses within several minutes (Witten et al., 2011; Ilango et al., 2014). However, animals show remarkably rapid reductions in response rate when the conditioned response is no longer reinforced (i.e., extinction) (Witten et al., 2011; Ilango et al., 2014). This set of observations is quite distinct from the food-reinforced response, which typically persists for a prolonged period of time during extinction. Therefore, we tested the hypothesis that photo-pulse trains inducing phasic excitation of DA neurons briefly potentiate the initiation of conditioned responses, and that the responses are regulated by little or no long-term motivation. In this light, we contrasted photo-pulse train reinforcement with food reinforcement.

## Materials and methods

### Animals

Adult male tyrosine hydroxylase (TH)::IRES-Cre knock-in mice (Lindeberg et al., 2004) crossed with C57BL/6j (The Jackson Laboratory, Bar Harbor, Maine) were used. Mice (weighing 24–35 g, 2–4 months old at the time of surgery) were group housed until surgery upon which they were individually housed in rooms maintained with a 12:12 light-dark cycle (lights on at 07:00 AM). Mice had free access to food and water outside of daily testing (30–50 min), with the exception of 6 mice (experiment 5 below) that were food-restricted in such a way that they received 1.5–3 g of rodent chow once a day to maintain 85% of their original weights. All procedures were approved by the Animal Care and Use Committee of the Intramural Research Program at National Institute on Drug Abuse and were in accordance with the Guide for the care and use of laboratory animals (National Research Council, 2011). All efforts were made to minimize suffering and the number of animals for research use.

### Viral vectors

NIDA Optogenetics and Transgenic Technology Core produced the serotype 1 adeno-associated virus (AAV1) encoding ChR2 and enhanced yellow fluorescent protein (EYFP) from plasmids obtained from the Stanford Optogenetics Innovation Lab (pAAV1-Ef1a-DIO-hChR2(H134R)-EYFP-WPRE-pA). The final viral concentration was 6.3 × 10^12^ viral genomes per ml.

### Construction of optical fibers

Standard hard cladding multimode fibers with the core size of 200 μm and numerical aperture of 0.37 (BFL 37–200, Thorlabs, Newton, NJ) were stripped off at the end and inserted into the 1.25 mm zirconia ferrule (MM-FER 2007C-2300, Precision Fiber Products) (Sparta et al., 2012). The fiber was cut approximately 6 mm in length and the tapered end was polished with different gradients of silicon carbide and aluminum oxide sheets. We used an optical-power meter to measure the intensity of laser-light output. Only fibers emitting concentric circles of light with 75% or higher light transmission were used for implantation. We recorded the percent of light transmission for each fiber and adjusted laser-light output intensities accordingly during experimentation.

### Stereotaxic surgery

Each mouse was anesthetized with a ketamine/xylazine mixture (80/12 mg/kg, i.p.) and received an injection of the AAV vector into the VTA (coordinates: AP 3.5 mm posteriorly from bregma, ML 0.5 mm laterally from midline, DV 4.0 mm ventrally from the dorsal surface of the brain). The AAV vector was microinjected in the volume of 1 μl over 10 min using a syringe pump. Then, an optic fiber aiming at 0.2 mm dorsal to the injection site was permanently secured on the skull using dental cement. A dust cap was placed at the tip of the implanted fiber to protect its surface when they were not in test chambers. After the surgery, mice were singly housed, and 2–4 weeks were given to allow gene expression before experiments were started.

### Apparatus

All behavioral experiments were conducted in standard operant conditioning chambers (ENV-307W: Med Associates, St. Albans, VT). Each chamber was equipped with two levers, two cue lamps and a house lamp. Mice were gently connected to the optic-fiber cable that was connected to a 473-nm DPSS laser (OEM Laser Systems, Bluffdale, UT) via an optical commutator. A computer interface system (Med Associates) coordinated pressing on the lever and a stimulus generator (Master 9, A.M.P.I, Jerusalem, Israel) that produced pulses and controlled the laser.

### Experiment1: Confirmation of phasic excitation of DA neurons by photo-pulse trains

Electrophysiological tests were performed in two mice. The administration of photo trains and recording of neural activity was accomplished by a screw-driven microdrive coupled with an optic fiber (105 μm in diameter) and 7 tetrodes (28 wires). Tetrodes were inserted into 7 individual polyamide cannulas (inner/outer diameters 100/160 μm, Ploymicro Technologies), and the polyamide cannulas were then glued on to the optic fiber. The tips of the tetrodes were separated from the tip of optic fiber by 300–500 μm. Each tetrode consisted of four 17-μm diameter platinum wires (90% Platinum 10% Iridium, California Fine Wire; with impedances of 1–2 MΩ for each wire). A similar optic-fiber/tetrode microdrive assembly was previously described (Cohen et al., 2012). Two weeks after surgery, electrode signals were screened daily as a function of photo stimulation in two unanesthetized, freely-moving mice. If neural activity responsive to photo stimulation was not detected, the microdrive was lowered by 50–100 μm daily. This procedure was repeated until we found neural signals responsive to photo stimulation. Neural signals were recorded using a Digital Lynx recording system (Neuralynx, Bozeman, MT). Spike signals were band-pass-filtered between 300–5000 Hz and digitized at 32 kHz. Spike signals were sorted offline using the Plexon OfflineSorter. Sorted neural spikes were processed and analyzed in NeuroExplorer (Nex Technologies, Madison, AL).

### Initial self-stimulation training

For behavioral experiments, we used three groups of mice that were trained to respond on the lever reinforced by photo-pulse trains exciting VTA DA neurons. We employed within-subjects designs, and each mouse received both control and experimental manipulations. For all the three groups, a press on the active lever was accompanied by the presentation of a discrete visual cue lasting 1 s during self-stimulation acquisition. The 1-s visual cue was provided to facilitate operant conditioning (Kish, 1966; Shin et al., 2010). However, to eliminate possible confounding effects (Shin et al., 2010; Keller et al., 2014), visual cue was not accompanied with lever-pressing in all other experiments described in the present study. In addition, for the purpose of simplicity, we only described the analyses of active-lever presses in all of the experiments described below, except the initial acquisition test (experiment 2), which will show that inactive-lever presses are low throughout the experiment and do not significantly change as a function of manipulation.

### Experiment 2: Replication of how mice acquire and extinguish self-stimulation behavior reinforced with phasic excitation of DA neurons

**Figure 1:**
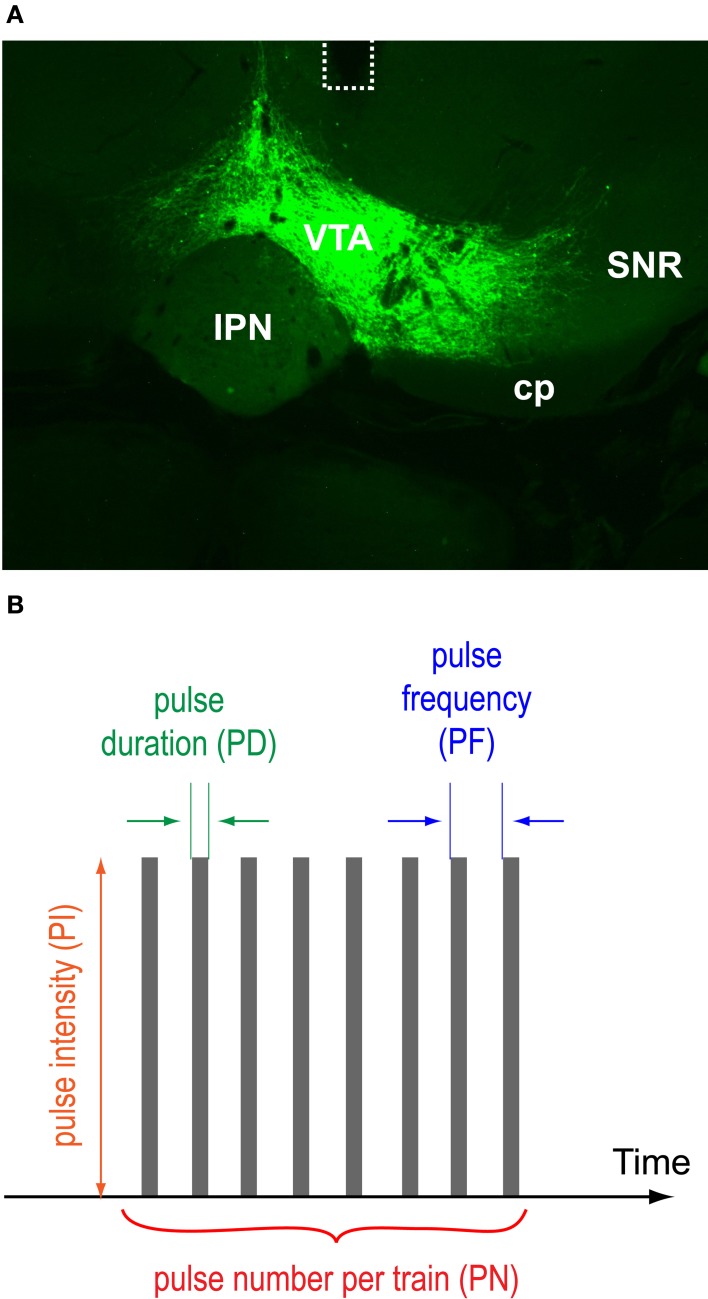
**VTA protein expression, optic fiber placements, and pulse-train parameters. (A)** A photomicrogram showing representative EYFP expression with the placement of an optic fiber (dotted line). Abbreviations: cp, cerebral peduncle; IPN, interpeduncular nucleus; SNR, substantia nigra pars reticulata; VTA, ventral tegmental area. **(B)** Pulse duration, intensity, frequency, and number of a photo-pulse trains varied.

The first group (*N* = 8) was experimentally naïve and was trained as described below with the photo-pulse train (Figure 1A) consisting of 1 ms pulse-duration (PD), 10 mW pulse-intensity (PI), 25 Hz pulse-frequency (PF), 15 pulse-number (PN). Their extinction-effect experiment used the photo-pulse train consisting of 10-ms PD, 10-mW PI, 25-Hz PF, 15 PN.

### Experiment 3: Effects of parametric manipulations of photo-pulse trains on self-stimulation

Group 1 mice were then tested with a continuous reinforcement (CR) procedure, where pressing on the lever was immediately reinforced by a photo-pulse train, and the next reinforcement was available as soon as the ongoing train ended. The level of each independent variable (PD: 1, 3, and 10 ms; PI: 0.1, 1, and 10 mW; PF: 8, 20, and 50 Hz) was changed every 15 min, testing 3 levels in ascending order in one session and then in reversed (descending) order in another session. Thus, effects of each parametric variable were tested over two 45-min sessions, and sessions were separated by 1–2 days. These parametric levels were obtained by consulting with previous studies that stimulated VTA DA neurons (Tsai et al., 2009; Adamantidis et al., 2011; Witten et al., 2011; Kim et al., 2012; Ilango et al., 2014).

Mice in the second group (*N* = 7) were initially used in another study described by Ilango et al (2014). These mice extensively received VTA photo-pulse trains (10-ms PD, 20-mW PI, 25-Hz PF, 15 PN) prior to the present experiments, and went through the acquisition of self-stimulation, operant extinction, re-acquisition, and place-preference tests, adding up to about 17–40 test sessions (30 min per session) before they were used for the experiments described below. The Ilango study (2014) describes the prior test procedures and histological data of group 2 mice. These mice were used to examine effects of pulse-number. They were tested for lever pressing reinforced on a CR schedule with photo-pulse train (PD: 10 ms; PI: 20 mW; PF: 25 Hz) of varying pulse-number: 0, 2, 4, 8, and 16 pulses. Each pulse's effects were examined in a 10-min period (or 2 bins of 5 min), and the pulses were tested in ascending order over a 50-min session. At the start of each period, mice received 5 “priming” trains (one train per s) consisting of the number of pulse that was tested for that period, to stimulate pressing. The number of presses during the 2nd bin of each 10-min pulse-period was used to represent the effect of the specific pulse-number. These counts were analyzed with a One-Way within-subjects ANOVA.

### Experiment 4: Effects of inter-reinforcement interval on self-stimulation

Group 2 mice were then used to examine effects of inter-reinforcement intervals. Four intervals were tested over five 10-min blocks in the order of 0, 1, 3, 10, and 0 s. The 0-s interval was repeated at the end, to show that mice were still capable of pressing at similar levels as the first 10-min period after a series of intervals. Effects of these intervals were examined with 4 different pulse numbers (2, 4, 8, and 16) per train (PD: 10 ms; PI: 20 mW; PF: 25 Hz) over 4 sessions, to determine how different values of reinforcer would interact with reinforcement intervals. Technically, the 0-s interval was interval schedules of 50–610 ms depending on how many pulses the reinforcer contained, since additional reinforcement was not provided during photo-pulse trains. For simplicity, we called it 0-s interval or CR schedule. Similarly, 1–10 s intervals were also slightly longer depending on the length of photo-pulse trains. The press count of the 2nd 5-min bin of each 10-min interval was analyzed with a 5_interval_ × 4_pulse−number_ within-subjects ANOVA.

### Experiment 5: Effects of ratio schedules on responses reinforced with food pellets or photo-pulse trains

The third group (*N* = 6) used for this experiment were initially used in still another study where they were trained to self-stimulate for photo trains (PD: 1 ms; PI: 10 mW; PF: 50 Hz; PN: 8) exciting VTA DA neurons on a CR schedule up to 12 sessions, and then they had received a bundle of electrodes implanted into the ventral striatum for recording, which was not performed in the present experiment. After the surgery, they were tested with VTA photo-pulse trains (PD: 1 ms; PI: 0.1–10 mW; PF: 8–50 Hz; PN: 8) for additional 8 sessions (30–60 min per session). Following these testing experience, they were used to examine effects of ratio schedules of reinforcement as described in the result section.

### Histology

After completion of the behavioral experiment, all animals were deeply anesthetized with a ketamine/xylazine mixture (80/12 mg/kg, i.p.) and intracardially perfused with ice cold 0.9% saline followed by 10% formalin. Brains were isolated and post-fixed in 10% formalin up to 24 h and cryoprotected in 30% sucrose solution until they sank to the bottom. Brains were coronally sectioned at 40 μm, and mounted sections were cover slipped with Mowiol 4–88 (Sigma-Aldrich) plus Vectashield mounting medium with DAPI nuclear counterstain (H-1200, Vector laboratories, Burlingame, CA). Optical fiber placements and EYFP expression were confirmed with fluorescent microscopy.

### Statistical analyses

All data pertaining to lever presses were square-root transformed, to minimize heterogeneous variances for parametric statistical tests (McDonald, 2009). Square-root transformed data were analyzed with the repeated measures of ANOVA using Statistica (version 6.1, StatSoft, Inc., Tulsa, OK). Significant effects were further analyzed by the Tukey's Honestly Significant Difference (HSD) *post-hoc* test.

## Results

To phasically stimulate VTA DA neurons, we injected a double-floxed inverted open reading frame (DIO) Cre-dependent AAV1 vector encoding ChR2 fused with EYFP into the VTA of TH-Cre transgenic mice and implanted an optic fiber just dorsal to the VTA for photo-pulse trains. Two to four weeks after the surgery, we examined effects of photo-pulse trains (473-nm wavelength) through the implanted optic fiber. Figure 1A displays a histological result showing the expression of EYFP and the placement of optic fiber of a representative mouse. Expression of EYFP and optic fiber placements were confirmed in all the mice we tested in this study.

### Experiment1: Confirmation of phasic excitation of DA neurons by photo-pulse trains

**Figure 2:**
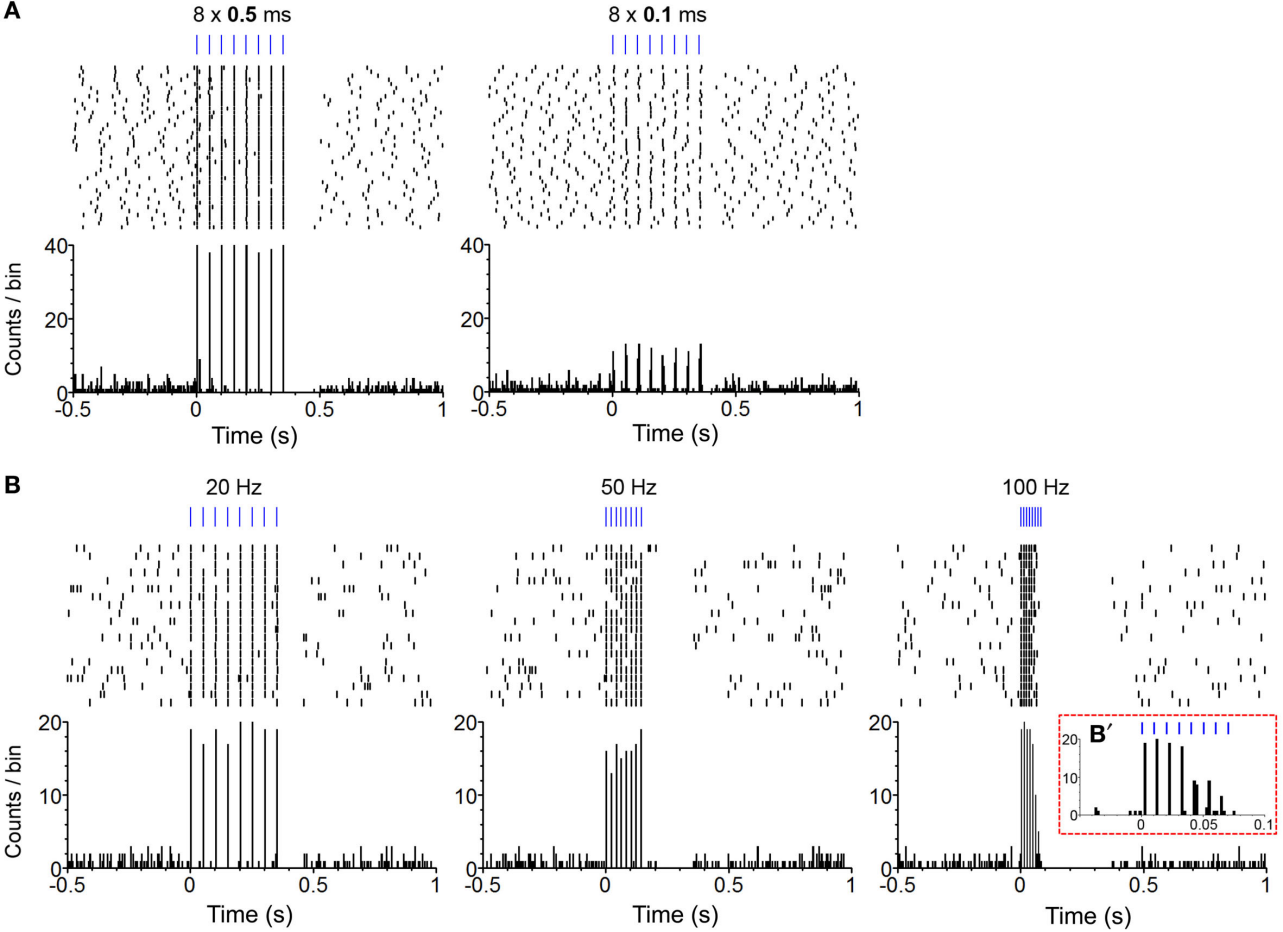
**Confirmation of phasic excitation of DA neurons by photo-pulse trains in freely-behaving mice. (A)** The perievent spike rasters (top) and histograms (bottom) show photo stimulation-evoked firing of a neuron. Experimenter administered 40 photo trains (PI: 0.2 mW; PF: 20 Hz; PN: 8) of the 0.5 (left) and 0.1 (right) ms pulse-durations with the inter-train intervals varying between 10 and 15 s. Bin = 5 ms. **(B)**. The perievent spike rasters (top) and histograms (bottom) show photo stimulation-evoked firing of a neuron. Experimenter administered 20 photo trains (PD: 1 ms; PI: 0.2 mW; PN: 8) of the 20- (left), 50- (middle), and 100-Hz (right) frequencies with the inter-train intervals varying between 10 and 15 s. Bin = 5 ms. **(B')** It shows an enlarged histogram for the 100-Hz train. Bin = 2 ms.

We confirmed in two mice that our photo-train administration procedure was capable of inducing phasic firing in ChR2-expressing (i.e., TH-positive, DA) neurons and modulating phasic firing as a function of change in pulse-train property (Figure 1B). In one mouse, we compared effects of pulse durations between 0.1 and 0.5 ms on neural response. The 0.5-ms pulse almost always excited the DA neuron, whereas the 0.1-ms pulse led to 10-15 action potentials out of 40 trials (less than 40% fidelity; Figure 2A). Three different frequencies of a pulse train were tested in a neuron of another mouse. The administration of an 8-pulse train at 20 or 50 Hz caused reliable excitation for almost every pulse (Figure 2B: left and middle panels), whereas 100-Hz trains reliably excited the neuron for the first 4 pulses, but failed to reliably excite it for the last 4 pulses of the train (Figure 2B: left panel insert). Previous electrophysiology experiments suggested that the success rate of triggering an action potential in DA neurons decreased markedly when frequency increased greater than 20 Hz, reaching only 50% of spike fidelity with the frequencies of 40–50 Hz (Tsai et al., 2009; Witten et al., 2011). Inconsistently, we found that spike fidelity with the 50-Hz train was as good as that of the 20-Hz in a single neuron. Note also that DA neurons became inactive immediately after the offset of photo-pulse trains, and the length of neural inactivity seems to have progressively increased as the train frequency increased (Figure 2B). Although these data in two DA neurons do not allow us to generalize how DA neurons respond to photo-pulse trains, the data confirm that our optogenetic-stimulation procedure induces phasic firing in neurons and seem to be in general agreement with what is known about how ChR2 responds to light (Nagel et al., 2003; Boyden et al., 2005) and how DA neurons fire (Tsai et al., 2009; Wang and Tsien, 2011; Witten et al., 2011).

### Experiment 2: Replication of how mice acquire and extinguish self-stimulation behavior reinforced with phasic excitation of DA neurons

**Figure 3:**
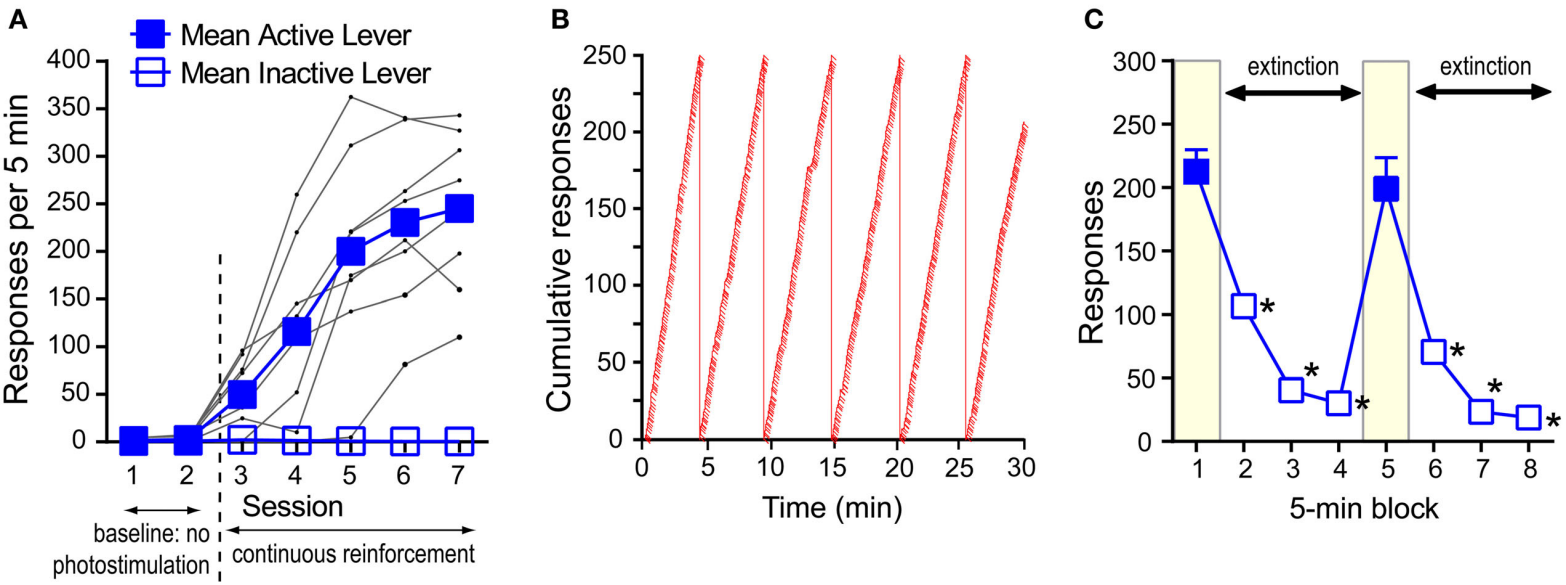
**Self-stimulation behavior with phasic excitation of DA neurons during acquisition and extinction. (A)** Mean rates of active- and inactive-lever presses and presses of individual mice (black dots) during acquisition. Active-lever values in sessions 3–7 were significantly greater than those of session 1 or 2 (*Ps* < 0.0005). **(B)** The cumulative presses of a representative mouse that responded at the rate of 243 per 5 min in session 7. When the cumulative lever-press count reaches 250, the count is reset at 0, and the angle of the cumulative-lever-press line indicates the rate of lever-press. Slashes indicate reinforcement incidents. **(C)** Rapid decrease in pressing during extinction. The data are mean with s.e.m. ^*^P < 0.0005, value significantly lower than that of block 1 or 5.

Experimentally-naïve mice (*n* = 8) were individually placed in an operant conditioning chamber equipped with two levers. During the first two sessions (30 min per session), a press on the “active” lever delivered the presentation of a 1-s visual cue just above the lever, but no VTA photo-pulse train, while pressing on the other “inactive” lever had no programmed consequence throughout this experiment. During the first two sessions, pressing on both levers remained relatively low (Figure 3A). In sessions 3–7, a press on the active lever delivered a 1-s visual cue and photo-pulse train (0.57 s in duration), and the next photo-pulse train became available for the taking as soon as the visual cue was turned off. The mice increased pressing on the active lever over sessions 3–7. A 2_lever_ × 7_session_ ANOVA revealed a significant lever × session interaction [*F*_(6, 42)_ = 49.12, *P* < 0.0001]. While presses on the active lever did not differ between sessions 1 and 2, response counts significantly differ between sessions 2 and 3 (*P* < 0.001), 3 and 4 (*P* < 0.05), and 4 and 5 (*P* < 0.005, Tukey's test). Response rates of the active lever began leveling out by sessions 5; presses in sessions 5–7 were significantly greater than those of sessions 1–4 (*P* < 0.0005) or inactive-lever presses of respective sessions (*P* < 0.0005, Tukey's test). Inactive-lever presses did not differ between sessions 1–7 (Tukey's test). Figure 3B shows the response pattern of a representative mouse that responded at the rate of 243 per 5 min in session 7. The data suggest that pressing on the active lever was so vigorous that the mouse took few breaks from pressing on the lever and maintained a relatively-constant response-rate throughout the 30-min session. By session 7, this response pattern was typical among the mice.

We then showed how quickly mice would change lever-press rates when pressing was no longer reinforced with photo-pulse trains (i.e., extinction). Effects of extinction were examined in a 40-min session consisting of two 20-min phases. In each phase, pressing on the active lever was reinforced with photo-pulse trains during the 1st 5-min block, followed by a 15-min extinction phase. Mice responded at a rate of over 200 presses during the 1st 5-min block and quickly decreased pressing during the extinction phase (blocks 2–4) (Figure 3C). After 5 min of no reinforcement, mice responded at rates of lower than 50 per 5-min (blocks 3 and 4). At the onset of the 2nd phase (i.e., block 5 in Figure 3C), mice received 5 “priming” trains administered one train per sec, and they quickly reinstated pressing on the lever to a level similar to that of the 1st block. Again response rates markedly decreased during the extinction phase (blocks 6–8) in a similar manner as in the first extinction phase (blocks 2–4). These effects are supported by a significant block effect [*F*_(3, 18)_ = 142.61, *P* < 0.0001 with a 2_phase_ × 4_block_ within-subjects ANOVA]. Although a phase × block interaction was not reliable, the second phase had slightly lower response rates than the first [a significant phase effect: *F*_(1, 6)_ = 15.17, *P* < 0.01].

### Experiment 3: Effects of parametric manipulations of photo-pulse trains on self-stimulation

**Figure 4:**
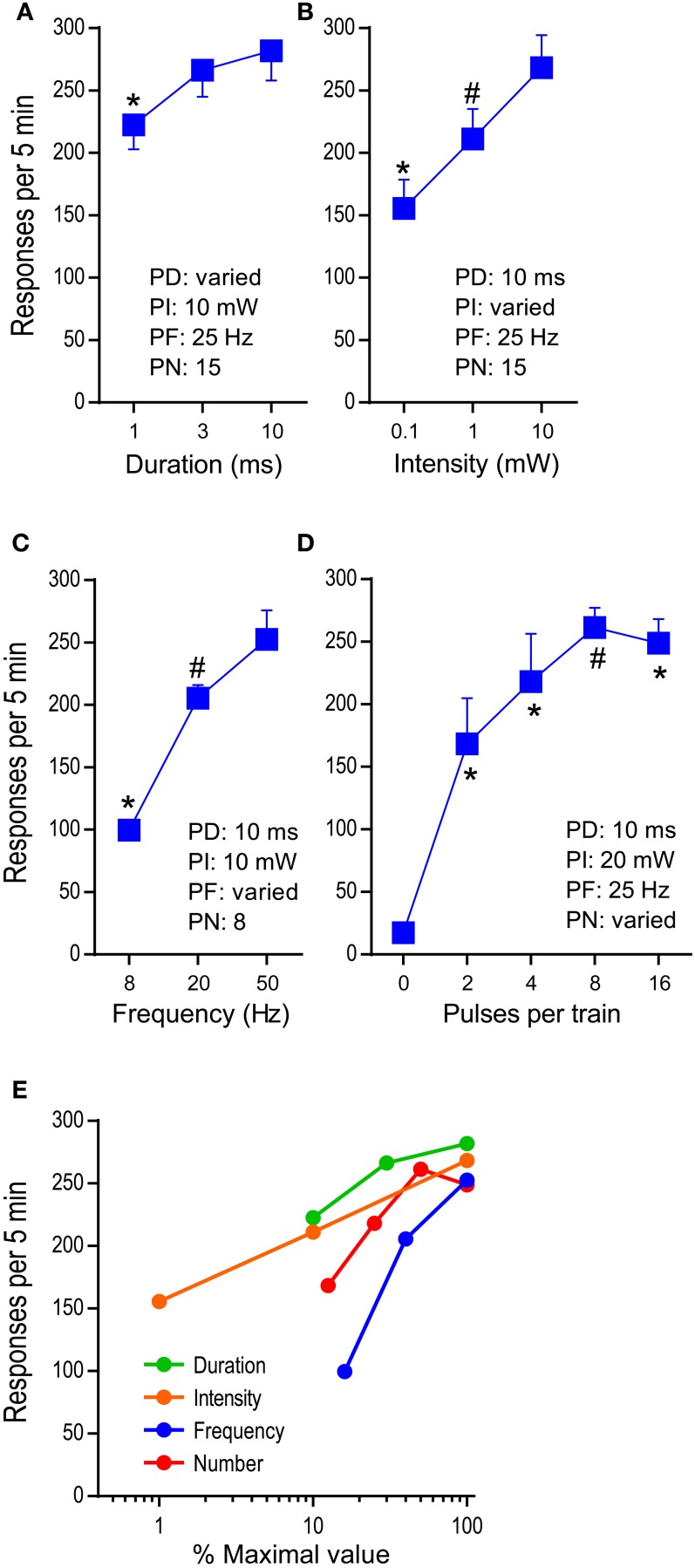
**Effects of parametric manipulations of photo-pulse trains on self-stimulation**. The data are mean with s.e.m. **(A)** Pulse duration was varied. ^*^P < 0.005, significantly lower than the 3- or 10–ms value. **(B)** Photo intensity was varied. ^*^P < 0.01, significantly lower than the 1- or 10-mW value; ^#^*P* < 0.05, significantly greater than the 0.1-ms value. **(C)** Pulse frequency was varied. ^*^P < 0.005, significantly lower than the 20- or 50-Hz values; ^#^*P* < 0.05, significantly greater than the 8-Hz value. **(D)** Pulse number was varied. ^*^P < 0.005, significantly greater than the 0-pulse value; ^#^*P* < 0.05, significantly greater than the 0- and 2-pulse values. **(E)** Summarized are effects of the factors: duration, intensity, frequency, and number. The maximal values of the factors (10 ms, 10 mW, 50 Hz, and 16 pulses, respectively) are set at 100%, and the rest of the values of each factor are transformed accordingly.

We examined effects of pulse-duration, pulse-intensity (measured at the tip of the ferrule that connects with the implanted optic fiber), pulse-frequency, and pulse-number per train in reinforcing behavior (Figure 1B). While no priming train was provided for descending-order sessions, for ascending-order sessions, mice received, at the onset of each 15-min period, 5 “priming” trains (with the rate of one train/s) whose parameters were assigned for that period, to reinstate pressing. The median lever-press count from 3 bins (5 min each) of each 15-min period was selected and analyzed with 2_test−order_ × 3_variable−level_ ANOVAs for active-lever presses. The main effect of test order was not significant for any of the variables; therefore, the data obtained with the ascending and descending orders are combined and presented together (Figures 4A–C). Lever-press rates were slightly reduced with the 1-ms pulse-duration than the 3- or 10-ms durations, and the 3-ms duration did not differ from the 10-ms duration [duration: *F*_(2, 14)_ = 15.66, *P* < 0.0005; Figure 4A]. All 3 pulse-intensities significantly differed from each other, with the 10-mW intensity being the most effective [intensity: *F*_(2, 14)_ = 21.09, *P* < 0.0001; Figure 4B]. The mice markedly reduced press rates with the 8-Hz train compared to the 20- or 50-Hz train [frequency: *F*_(2, 14)_ = 100.05, *P* < 0.0001; Figure 4C].

We then examined effects of pulse-number per train (PN: 2, 4, 8, and 16) on lever pressing, using the second group of mice (*n* = 7) described in the method section. The mice maintained low press rates during the 0-pulse (i.e., no stimulation) period and markedly increased pressing when it was reinforced with 2-pulse train or greater [pulse: *F*_(4, 24)_ = 33.49, *P* < 0.0001]. The 8-pulse train was significantly more effective than the 2-pulse, but not the 4-, or 16-pulse train (Figure 4D).

Figure 4E summarizes how lever-press rates were altered by pulse-duration, intensity, frequency, and number. The manipulations of various properties of photo-pulse trains readily alter their efficacy, leading to changes in lever-press rate. In addition, we should point out that despite of repeated test sessions, photo-pulse trains of DA neurons produced comparable data when the same parameters of photo-pulse trains was examined between sessions, as we observe in this experiment and in our previous study (Ilango et al., 2014). Therefore, the repeated-subjects design of experiments is generally effective method for the investigation involving stimulation manipulations on self-stimulation.

### Experiment 4A: Effects of inter-reinforcement interval and pulse number on self-stimulation

The manipulation of reinforcement schedules produces a wide range of change in behavior reflecting emotional and motivational processes (Ferster and Skinner, 1957). We examined effects of inter-reinforcement interval schedules, in which mice had to wait a given interval of time measured from the preceding reinforcement before their lever-pressing was reinforced.

**Figure 5:**
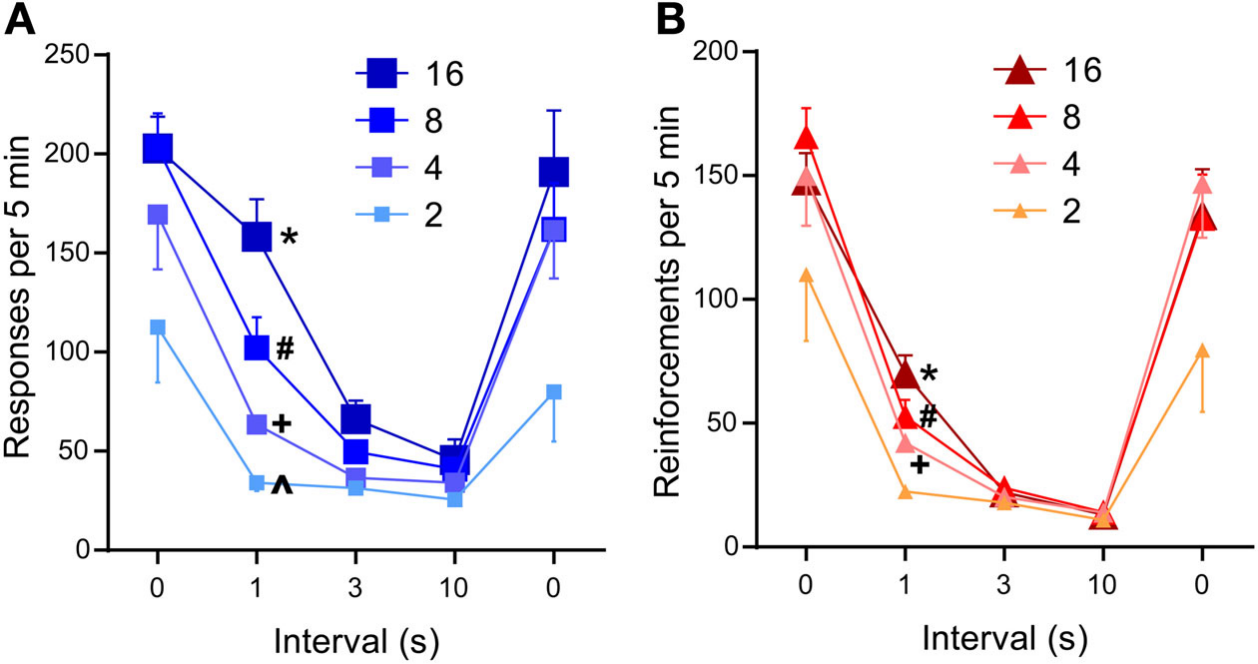
**Effects of inter-reinforcement interval on self-stimulation. (A)** Mean lever-press rates are shown as a function of inter-reinforcement interval and pulse number. All 0-s interval values were significantly greater than respective 3- or 10-s interval values (*Ps* < 0.001). ^*^P < 0.001, significantly greater than its 3- and 10-s values, but not from its 0-s value; ^#^*P* < 0.05, significantly greater than its 3- and 10-s values, but lower than its 0-s value; ^+^, ^∧^*P* < 0.001, not significantly different from its 3- and 10-s values, but lower than its 0-s value **(B)**. Mean reinforcement rates are shown as a function of reinforcement interval and pulse number. All 0-s interval values were significantly greater than respective 1-, 3-, or 10-s interval values (*Ps* < 0.001). ^*^P < 0.001, significantly greater than its 3- and 10-s values; ^#,+^*P* < 0.01, significantly greater than its 10-s values.

The 1-s interval was sufficient to significantly reduce lever-press rates compared to those of 0-s interval (*P* < 0.0005, Figure 5A), while the 3- or 10-s interval diminished lever-press rates [*P* < 0.0005, Tukey's test after a significant interval effect, *F*_(4, 24)_ = 44.41, *P* < 0.0001]. This result needs to be considered in light of the fact that the number of reinforcements obtainable per unit of time becomes fewer as reinforcement interval increases (Table 1). Because mice responded at relatively fast rates, the reduced numbers of pressing can be explained by the reduced availability of reinforcements.

**Table 1:** **Dependency of the numbers of photo-pulse trains obtainable per 5 min on photo-pulse trains durations and inter-reinforcement intervals**.

**Photo-pulse trains**		**Inter-reinforcement interval (s)**			
**Pulse #**	**Duration (ms)**	**0**	**1**	**3**	**10**
16	610	491	186	83	28
8	290	1,034	232	91	29
4	130	2,307	265	95	29
2	50	6,000	285	98	29

However, this reduced reinforcements explanation is not consistent with a significant interaction between reinforcement interval and pulse number [*F*_(12, 72)_ = 4.18, *P* < 0.0001], which shows opposite effects that this explanation predicts. Lever-press rates maintained with 16-pulse trains did not significantly differ between the 1-s and the 0-s interval schedules; the 8-pulse train significantly decreased press rates during the 1-s interval than the 0-s interval (*P* < 0.0005), while maintaining significantly greater press rates during the 1-s than the 3- or 10-s interval (*Ps* < 0.05 and 0.001, respectively); the 4- and 2-pulse train diminished press rates with the 1-s interval, which did not differ from those of the 3- or 10-s interval (Figure 5A). Therefore, graded effectiveness of 4-, 8-, and 16-pulse trains was apparent with the 1-s interval, effects that are not consistent with the fewer reinforcements explanation. Similar effects of pulse and interval were found on reinforcement incidents (Figure 5B): interval effect on reinforcement incidents is more robust than that of lever presses, while interval-pulse interaction is less striking [interval: *F*_(4, 24)_ = 80.44, *P* < 0.0001; interval × pulse interaction: *F*_(12, 72)_ = 2.96, *P* < 0.005]. In summary, even though more trains were available for the 2- and 4-pulse trains than the 16-pulse train, the 2- and 4-pulse trains did not maintain response rates greater than that of the 16-pulse train. Therefore, the reduction of response rates must be largely caused by the longer inter-reinforcement interval reducing motivation for pulse trains.

### Experiment 4B: Effects of inter-reinforcement interval and pulse number on the first lever press after reinforcement

**Figure 6:**
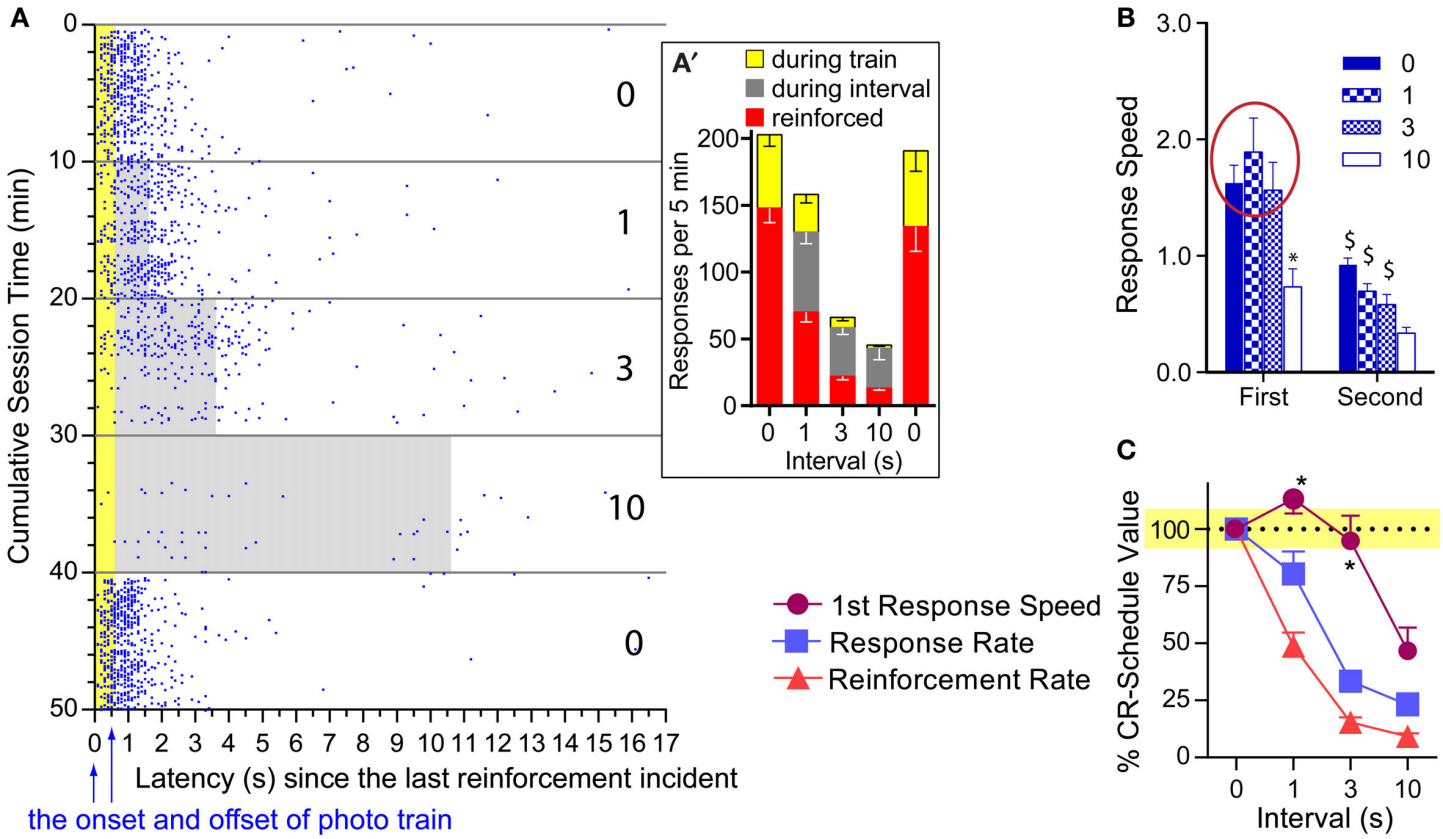
**Effects of pulse and interval on the first response after reinforcement. (A)** Lever-press incidents with the 16-pulse train are plotted in relation to the last reinforcement incident and train duration. When presses shown in blue dots were emitted during the train duration (shown in yellow area) or during inter-reinforcement interval (gray area), they were not reinforced. The number on the right indicates inter-reinforcement intervals for the 10-min periods. **(A')** Mean presses that were reinforced and occurred during ongoing trains and intervals. **(B)** Mean lever-press speeds (1/latencies) of first and second presses after reinforcement incidents as a function of pulse and interval. ^*^P < 0.005, significantly slower than the 0-, 1-, and 3-s interval values. ^$^*P* < 0.005, significantly slower than its first response speed. **(C)** Mean percent of CR-schedule values. ^*^P < 0.005, significantly greater than lever-press rate or reinforcement rate value for respective interval.

One notable feature of photo-pulse train reinforcement is that the mice responded on the lever even during ongoing photo-pulse trains. Figure 6A plots the lever-press events of a representative mouse in relation to reinforcements and intervals when tested with 16-pulse trains. Lever pressing during ongoing trains (610 ms in duration) was not reinforced with additional photo-pulse trains. Despite this, this mouse and the others responded often during ongoing trains (Figure 6A′). This observation is consistent with our hypothesis that photo-pulse trains potentiated the initiation of *conditioned* approach response. However, this observation is also consistent with the notion that mice responded during the photo-pulse trains because mice responded on the lever so fast that responding had its *momentum*, which caused responding even during the non-reinforced period. The results described below are more consistent with the response initiation hypothesis than the momentum explanation.

We compared speeds between the first and the second lever-presses as a function of interval for each pulse, using 2_press−order_ × 4_interval_ ANOVAs. A significant press-order × interval interaction was found for the 16-pulse train [*F*_(3, 18)_ = 6.23, *P* < 0.005; Figure 6B: right panel]. The 1- and 3-s intervals did not significantly reduced the first press speed, while the 10-s interval did; and the speeds of second press was significantly reduced for all intervals compared to those of the first (Figure 6B: left panel). To determine how differently overall response rates, reinforcement rates, and first lever-press speeds were altered by interval schedules, we converted the 1-, 3-, and 10-s interval values of these variables with respect to their 0-s interval values as being 100% (Figure 6C). Normalized values were analyzed with a 3_variable_ × 3_interval_ ANOVA. The 16-pulse data revealed a significant variable x interval interaction, [*F*_(3, 18)_ = 3.56, *P* < 0.05]. Lever-press rates and reinforcement rates had lower values than first lever-press speeds for the 1- (*P* < 0.05 and *P* < 0.0005, respectively) and 3-s (*P* < 0.0005 and *P* < 0.0005, respectively) intervals (Figure 6C: left panel). The lever-press speed was also significantly different from reinforcement rate during the 10-s interval schedule (*P* < 0.05). Lever-press rates did not differ from reinforcement rates at any interval (Supplemental Figure 1 provides comparisons for the 8- and 4-pulse trains). Therefore, the clear dissociation between first lever-press speeds and response rates supports the response initiation hypothesis, but not the momentum explanation.

### Experiment 5: Effects of ratio schedules on responses reinforced with food pellets or photo-pulse trains

The above observation about interval schedules suggest that timely reinforcement with DA signals is critical in maintaining fast, constant lever-pressing, and any schedule that prevents mice from obtaining timely reinforcement will disrupt pressing. To further confirm this notion, we examined how mice would respond for photo-pulse trains when challenged by fixed-ratio (FR) schedules in which a lever-press was reinforced after a completion of a fixed number of presses counted from the last delivery of reinforcer. Effects of FR on self-stimulation were contrasted with those on food-seeking. The third group of mice described in the method section were trained to respond for food pellets on various FR schedules first and then re-trained to respond for photo-pulse trains exciting DA neurons.

**Figure 7:**
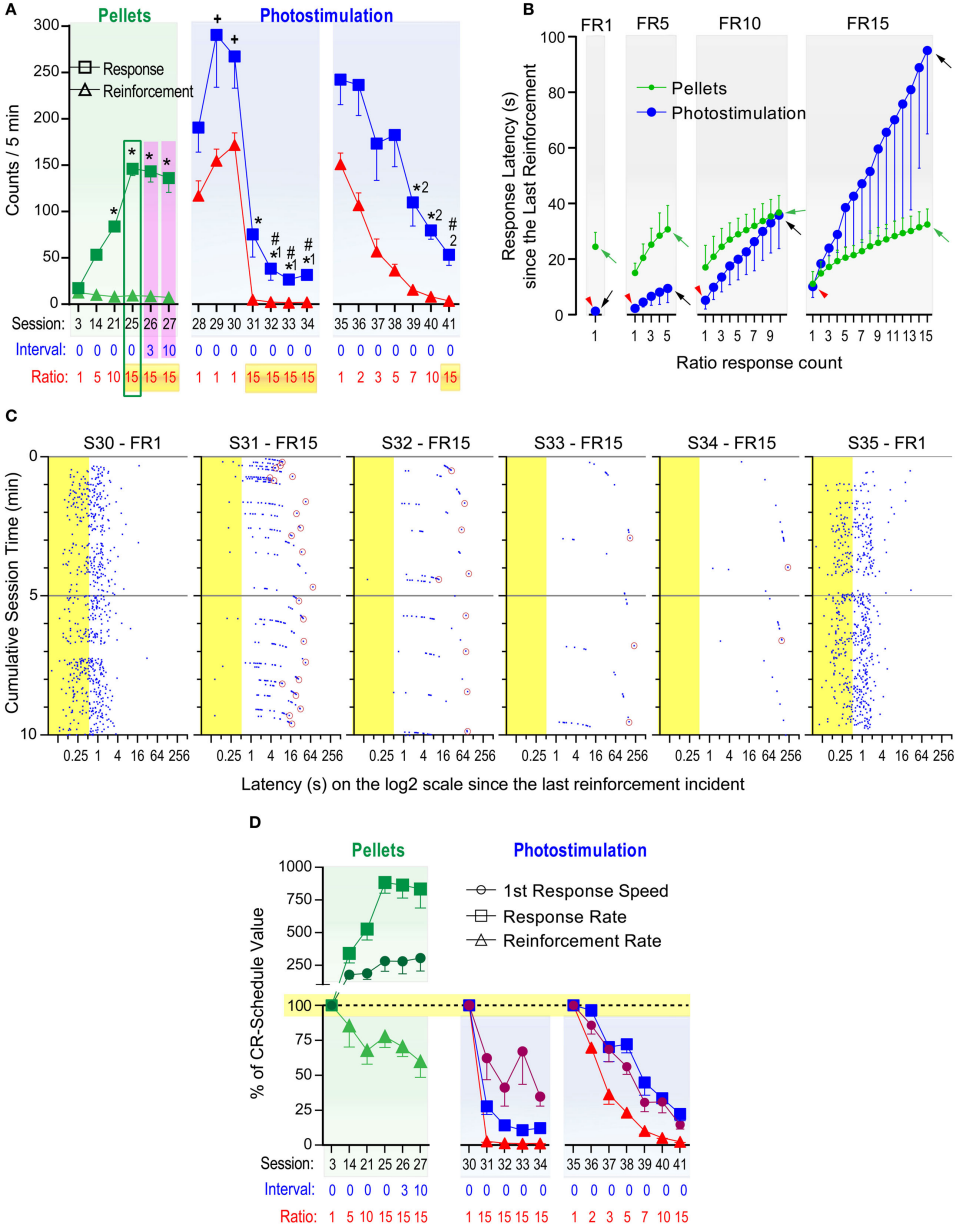
**Effects of fixed ratio schedules on responses reinforced with food pellets or photo-pulse trains**. Fixed-ratio (FR) schedules reinforce lever pressing after a completion of a fixed number of presses counted from the last delivery of reinforcer. **(A)** Mice were trained to respond for food pellets over 27 sessions. ^*^P < 0.005, significantly greater than the session-3 value. During sessions 28–41, pressing was reinforced with VTA photo-pulse trains. ^+^*P* < 0.05, significantly greater than the session-25 value; ^*1^*P* < 0.005, significantly lower than the session-28 value; ^#^*P* < 0.005, significantly lower than the session-25 value; ^*2^*P* < 0.005, significantly lower than the session-35 value. **(B)** Mean lever-press latencies (s) as a function of lever-press count in FR schedules and reinforcers. Red arrowheads indicate the latencies to initiate leverpressing for photostimulation. Green and black arrows indicate the latencies to complete FR requirement for pellets and photostimulation, respectively. **(C)** Lever-press incidents during the first 10 min block during sessions 30–35. Notice that they are plotted on a log^2^ scale (x-axis). Presses shown in blue dots occurring during the train duration (shown in yellow area) were not reinforced. Every lever-press occurring after each pulse train was reinforced for FR1 session, while for FR15 sessions, only the 15th lever-press (indicated by a maroon circle) occurring after each pulse train was reinforced. **(D)** Mean percent of CR-schedule values. ^+^*P* < 0.05, significantly greater than reinforcement rate, but not response rate. ^*^P < 0.05, significantly different from both reinforcement and response rates.

Significant sessions effects were found following one-way within-subjects ANOVAs on lever-press and reinforcement rates with 20 selected sessions consisting of 6 food and 14 photo-pulse train sessions [*F*_(19, 95)_ = 25.67, *P* < 0.0001 and *F*_(19, 95)_ = 125.83, *P* < 0.0001, respectively; Figure 7A]. The same lever that had produced VTA photo-pulse trains in the previous study also delivered pellets, and these mice quickly learned to respond for pellets and displayed stable pressing for pellets during the first 3 sessions, although pellet reinforcement on a CR schedule kept lever-press rates low (Figure 7A: session 3, left panel). We used FR schedules in the following sessions, to examine how FR challenges alter lever pressing. The mice were initially trained to respond 5 times on the lever for a pellet (i.e., FR5) for 11 sessions, then on a FR10 for 7 sessions and on a FR15 for 4 sessions. The mice learned to respond about 150 times per 5 min on the FR15 schedule in session 25, while earning similar numbers of pellets to those of sessions 3 (CR schedule; Figure 7A: left panel). Figure 7B depicts that the mice obtained pellets with the mean inter-reinforcement interval of 24 s on the CR schedule, and they increased rates of pressing as response-ratio requirement increased for a pellet in such a way that they maintained similar inter-reinforcement intervals (green arrows) across the different ratio schedules. When they were tested with FR15 combined with fixed-interval (FI) schedules of 3 and 10 s in sessions 26 and 27, respectively, they displayed numbers of lever presses and reinforcements similar to those of session 25 (Figure 7A: left panel). These results confirm that mice can regulate lever-press rates in response to ratio challenges to obtain food at a constant rate.

After session 27, the mice were no longer food-restricted and received VTA photo-pulse trains (PD: 10 ms; PI: 20 mW; PF: 25 Hz; PN: 15), instead of pellets, as reinforcer. While lever-press rates in sessions 29 and 30 on a CR schedule were greater than that of pellets in session 25 (Figure 7A: left panel), lever-press rates plummeted when the mice had to earn photo-pulse trains on the FR15 schedule in sessions 31–34 (Figure 7A: middle panel). Lever-press rates of sessions 31–34 were significantly lower than those of session 25, and repeated testing with the FR15 schedule tended to worsen self-stimulation performance until session 35 when the CR schedule was reinstated (see Supplementary Figure 2). Figure 7C depicts pressing patterns of the best self-stimulator (with respect to lever-press rate) and shows that its performance deteriorated over sessions 31–34, while it recovered in session 35 with the CR schedule to a similar level as that in session 30. Lever-press rates of this and other mice during 31–34 were reduced to similar levels as those we observed in other groups on extinction or interval schedules of 3 and 10-s (Figures 3, 5). Therefore, repeated testing with FR 15 did not appear to help improve self-stimulation performance even though the same animals had responded at a high level for pellet reinforcer with the same schedule.

We then examined effects of a gradual increase in response requirement over sessions on lever pressing reinforced by photo-pulse trains. Their lever-press rates significantly decreased as the lever-press ratio increased over the sessions (Figure 7A: right panel). Specifically, as the response-ratio requirement increased, lever-press intervals increased, resulting in longer and longer inter-reinforcement intervals (black arrows in Figure 7B). Thus, we did not detect evidence that mice maintained certain reinforcement rates with excitation of DA neurons across different FR schedules like they did with food pellets.

To determine how differently overall response rates, reinforcement rates, and first lever-press speeds were altered by FR schedules, we converted the values of these variables with respect to FR1 values as being 100%. Specifically, the values of sessions 14, 21, 25, 26, and 27 were normalized with respect to those of session 3 for the pellet reinforcement experiment (Figure 7D: left panel). A significant variable × session interaction was found with a 3_variable_ × 5_session_ ANOVA for pellet reinforcement [*F*_(8, 40)_ = 12.02, *P* < 0.001]. The response-ratio schedules involving pellets had opposing effects between lever-press and reinforcement rates, and affected them differently for all sessions (14, 21, 25, 26, and 27). While the speeds of the first presses and lever-press rates increased, lever-press rates increased to greater degrees than the first lever-press speeds for sessions 21, 25–27. The response-ratio schedules tended to increase lever-press speeds and decrease reinforcement rates for sessions 25–27.

Similarly, the photo-pulse trains data in sessions 30–34 were analyzed by a 3_variable_ × 4_session_ ANOVA with session-30 values being 100% (Figure 7D: middle panel). A significant variable effect was found [*F*_(2, 10)_ = 26.66, *P* < 0.005]. The FR15 schedule reduced first lever-press speeds less severely than lever-press rates (*P* < 0.005) and reinforcement rates (*P* < 0.001), and reduced similarly between lever-press and reinforcement rates (Tukey's test). The photo-pulse trains data in sessions 35–41 were analyzed by a 3_variable_ × 6_session_ ANOVA with session-35 values being 100% (Figure 7D: right panel). A significant variable and session effects, but not an interaction, were found [*F*_(2, 10)_ = 81.54, *P* < 0.0001 and *F*_(5, 25)_ = 31.24, *P* < 0.0001, respectively]. The response-ratio schedules reduced reinforcement rates more severely than first lever-press speeds (*P* < 0.005) and lever-press rates (*P* < 0.005), and first speeds than lever-press rates (*P* < 0.05, Tukey's test). Thus, an important difference in this analysis from the one for sessions 30–34 is that speeds were affected more than lever-press rates throughout sessions 35–41. This suggests that repeated experience with high ratio schedules diminished brief potentiating effect of photo-pulse trains on conditioned response.

## Discussion

### Effects of parametric manipulations on self-stimulation rates

The evidence that we consider below suggests that the parameters of photo-pulse trains that support fast, constant pressing probably excite DA neurons in such a manner that they significantly increase DA concentrations at projection regions. In addition, efficacy of photo-pulse trains in reward depends on how well photo-pulse trains interact with physical property of ChR2 and that of DA neurons, and whether it recruits a large population of DA neurons. Factors such as the density of ChR2 expression in each neuron should make a significant difference in stimulation efficacy between subjects and studies; because such factors are beyond the scope of the present study, we do not discuss them.

Reinforcement efficacy of pulse duration critically depends on how quickly ChR2 responds to light, allowing cations to enter the cell. ChR2 is capable of responding to light for maximal intracellular conductance within 0.5 ms (Bamann et al., 2008). Consistent with this observation, our preliminary investigation in a single neuron showed that the 0.5-ms pulse triggered action potential at almost 100%, while 0.1-ms pulse at about 50% (Figure 2B). We did find, however, 3- and 10-ms pulses were more effective in increasing self-stimulation rates than a 1-ms pulse (Figure 4A). There should not be any difference between them when the 0.5-ms pulse is sufficient in activating ChR2 at 100%. It is likely that 3- and 10-ms pulses can recruit more neurons by activating ChR2 at distal sites, where the 1-ms pulse is too short to deliver enough light energy for action potentials. That is, the pulse duration factor may interact with the distance factor in activating ChR2.

Photo intensity is known to exponentially decrease as the distance between its source and target increases. Therefore, the number of neurons affected by light is thought to depend on intensity. Consistently, we found that self-stimulation performance became better and better as the photo intensity increased from 0.1 to 1 to 10 mW (Figure 4B). Likewise, voltammetry study found that as the intensity increases, DA is released more in projection regions (Bass et al., 2010).

The pulse frequency factor exquisitely affected self-stimulation performance. Pulse frequency is important because it controls phasic firing rates of DA neurons and interacts with the DA uptake process that keeps extracellular DA concentration in check. That is, the higher the frequency that DA neurons fire, the greater the DA concentration becomes, because DA release outperforms the uptake process. However, as frequency increases, each subsequent pulse loses its fidelity to excite neurons. Consistent with our single-unit data, we observed the 50-Hz train was significantly more effective than the 20-Hz in self-stimulation (Figure 4C), and our behavioral results are consistent with those reported by a recent self-stimulation study (Rossi et al., 2013). If spike fidelity of a 50-Hz train is 50%, a 50-Hz train would trigger about the same number of action potentials as the 20-Hz train; therefore, it was expected to see a similar self-stimulation rate between 20- and 50-Hz trains. The discrepancies may be resolved by considering the contributions of other factors. A voltammetry study suggests that the efficacy of frequency on spike fidelity depends on pulse duration. A pulse train with 20-ms pulse duration tended to decrease DA release when delivered at higher frequencies than 20 Hz, while a 4-ms pulse-duration train increased DA release up to 50 Hz and decreased it at higher frequencies (Bass et al., 2010). Caveats include recording and stimulation sites. Bass et al performed voltammetry in the dorsal striatum with the stimulation of substantia nigra DA neurons. Another important factor is the recording condition. Our frequency experiment on DA neuron spikes used a pulse duration of 1 ms recorded in an unanesthetized, unrestraint mouse, while the studies (Tsai et al., 2009; Witten et al., 2011) that reported 50% of spike fidelity with the frequencies of 40–50 Hz used 15- and 5-ms pulse durations, respectively, in slice recording conditions.

Pulse number had relatively small effects on lever-press rate. Given the voltammetry finding that as the pulse number increases, extracellular DA concentration increases (Bass et al., 2010; Witten et al., 2011), we expected to see large effects between 2, 4, 8, and 16 pulses. The 4-pulse train was almost as effective as the 8- or 16-pulse train (Figure 4D). The lack of difference between these is most likely explained by a ceiling effect in that mice appeared to self-stimulate as fast as they could with stimulation of 4 and greater pulses. Consistently, when challenged by 1-s interval schedule, the 16-pulse train supported greater lever-press rates than lower pulse trains.

### Phasic excitation of DA neurons potentiate the initiation of approach behavior

Previous studies noted that DA concentrations in the ventral striatum phasically increase during ongoing approach response that earns cocaine (Phillips et al., 2003), VTA stimulation (Cheer et al., 2007), or food (Roitman et al., 2004). It is tempting to interpret that DA releases coinciding with approach responses actually help to drive the behavior. Our finding that phasic excitation of VTA DA neurons potentiates the initiation of conditioned approach behavior (Figure 6C) provides direct evidence that indeed, phasic excitation of DA neurons, which results in phasic DA release in the ventral striatum (Wightman and Robinson, 2002), potentiates the initiation of conditioned approach behavior. Our data show that potentiated lever-pressing is not consistent with the explanation involving a motor reflex since its effect dissipated as inter-reinforcement interval increased (Figure 6B; Supplemental Figure 1A). It is not momentum either, since the 16-pulse train maintained the same initiation speeds between the 0- and 3-s intervals when overall rates of responding with the 3-s interval was reduced to 30% of the 0-s interval (Figure 6C). Moreover, it is a conditioned approach response, since mice learned not to respond with vigor after repeated training with large ratio schedules of reinforcement (Figures 7C,D and Supplemental Figure 2).

The present study also provides insight on other functional properties of phasic excitation of DA neurons. There appear to be, at least, two distinct mechanisms to control rates of conditioned lever-pressing reinforced by phasic excitation of DA neurons. We initially observed that mice needed many repetitions of response-reinforcer experience to acquire a high response rate (Figure 3A). This observation is consistent with the notions that many repetitions are needed to form and refine new skillful responses in an environment-dependent manner (Ferster and Skinner, 1957) and to form long-term memory concerning the associative structure of the environment and its action, i.e., associative learning, to maximize the procurement of reinforcers through “trial-and-error” processes (Hull, 1952; Sutton and Barto, 1998). Thus, response-contingent excitation of DA neurons triggers long-term changes pertaining to learning. In addition, phasic firing of DA neurons appears to have motivational effects. After acquiring self-stimulation, their response rates dropped from the high asymptotic level to a low baseline level during extinction in just several minutes and from the low to high levels during reacquisition in just a few seconds (Figures 3C, 6A). Our observations suggest that DA signals potentiated the initiation of conditioned lever-pressing, an effect that lasted for about 1 s (Figure 5A) and lacked long-term motivational control (Figure 7B). This property of DA signals most likely plays a critical role in fast, constant self-stimulation on a CR schedule and rapid reduction of self-stimulation upon relatively minor interval- or ratio-schedule challenges.

We have also observed that distinct regulations of behavior reinforced by DA neuron excitation and food pellets. Most notably, pellet reinforcement did not potentiate presses just after reinforcements, and it instead inhibited them for a few seconds (Figure 7B). This is not surprising as animals take time to consume earned food before next responses. On the other hand, photo-pulse trains exciting DA neurons did not distinguish between approach and consummatory responses or inhibit approach behavior upon the delivery of the reinforcer; instead, it facilitated pressing in a short-lasting manner (Figures 6A–C). Another striking feature of food reinforcement was that it regulates approach behavior in a long-term frame, a regulatory system that behavioral scientists referred to as “primary drive” or “primary motivation.” The mice were able to control pressing to obtain pellets at a constant interval, i.e., every 25–35 s when challenged with FR schedules; on the other hand, the same mice did not display such regulated responding with photo-pulse trains when challenged by the same environmental conditions (Figure 7B). Similarly, when challenged with interval schedules, mice working for photo-pulse trains fail to show persisting lever-pressing on the order of seconds (Figure 5A). Therefore, phasic excitation of DA neurons neither inhibited its approach behavior for consummatory processes nor supplied any long-term motivation.

Apparent lack of long-term motivation for phasic excitation of DA neurons raises interesting issues with respect to its role in addiction. DA has been implicated in various addictive behaviors, most strongly with abused drugs. It seems to be difficult to reconcile the role of DA in addiction with the rapid decline in photo-pulse reinforced response during extinction. Future research may address whether mere phasic excitation is sufficient to recruit addictive processes. Indeed, drugs can activate DA transmission for hours at a time before going back to a baseline level (Di Chiara and Imperato, 1988). It may also be important for environmental stimuli to be paired with phasic excitation of DA neurons, so that conditioned stimuli gain control over behavior (Flagel et al., 2011).

In summary, we found that phasic excitation of DA neurons potentiates the initiation of approach behavior, and this motivational effect quickly diminishes. The study provides direct evidence for the role of phasic excitation of DA neurons in motivation.

### Conflict of interest statement

The authors declare that the research was conducted in the absence of any commercial or financial relationships that could be construed as a potential conflict of interest.
