# Supplementary material for: Phasic excitation of ventral tegmental dopamine neurons potentiates the initiation of conditioned approach behavior: parametric and reinforcement-schedule analyses
Source: Front Behav Neurosci. 2014 May 6;8:155. doi: 10.3389/fnbeh.2014.00155 (PMC4018564; doi:10.3389/fnbeh.2014.00155)
Supplement: Supplementary file 1 [file DataSheet1.DOC]

Supplementary Figure 1


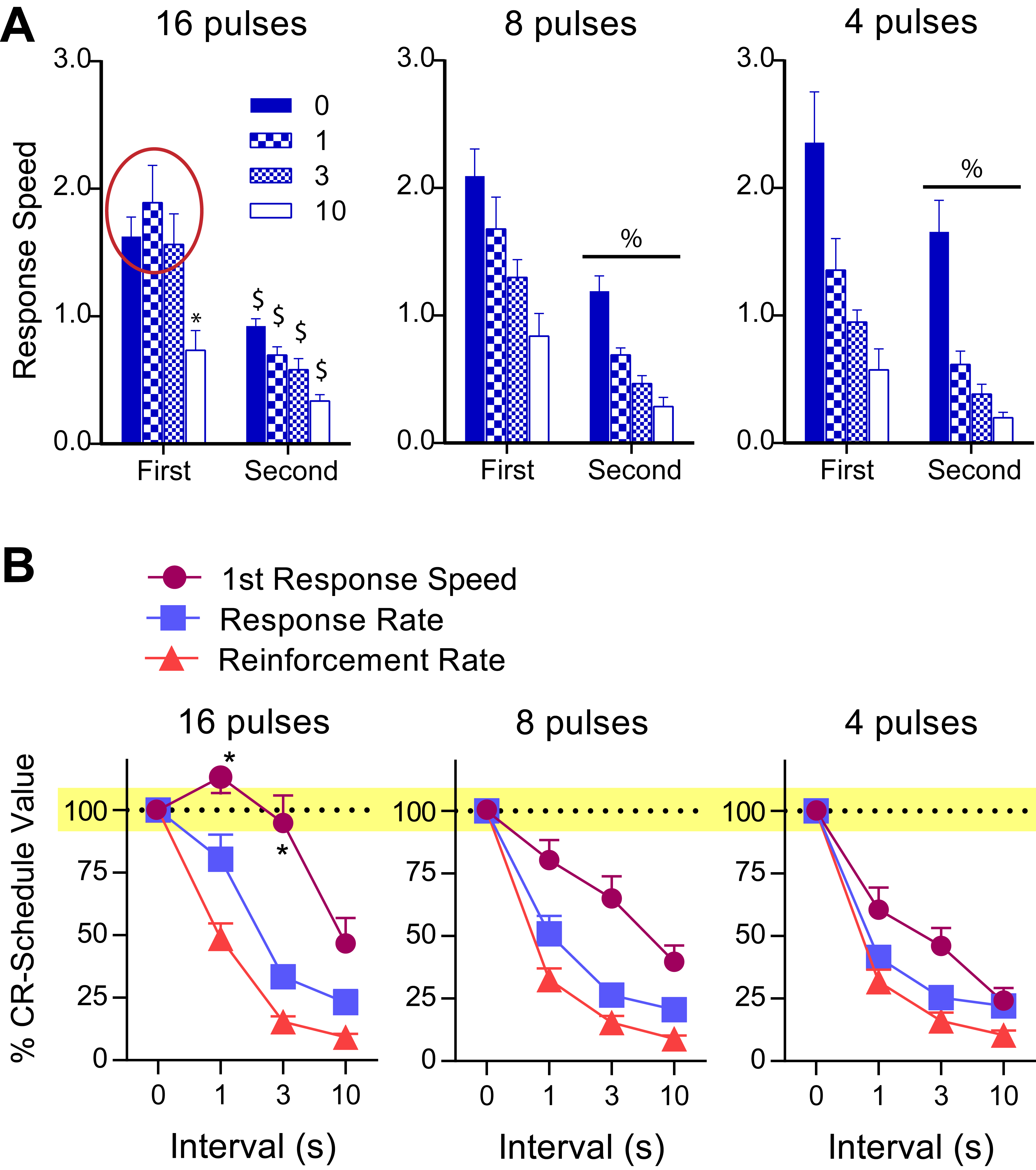


**Effects of pulse and interval on the first response after reinforcement**

**(A)** Mean lever-press speeds (1/latencies) of first and second presses after reinforcement incidents as a function of pulse and interval. As described in the experiment 4b section of the result section, we compared speeds between the first and the second lever-presses as a function of interval for each pulse, using 2_press-order_ x 4_interval_ ANOVAs. It should be noted that we were able to obtain second lever-press speeds only when first presses were not reinforced. Consequently, fewer data for second press speeds were available than first press speeds especially for the 0-s interval schedule. The data of 2-pulse trains were not analyzed, because the mice rarely emitted the second press with the 2-pulse train during the 0-s interval; each 2-pulse train only lasted 50 ms and the 50-ms period was not long enough to have an unreinforced lever-press. While the results for the 16-pulse train were described in Figure 6, the same panel is shown in the left for easy comparison with the 8- and 4-pulse train data (middle and right panels, respectively). ANOVAs for the 8- and 4-pulse trains revealed significant press-order effects (*F*_1_,_6_ = 84.19, *P* < 0.0001 and *F*_1_,_6_ = 19.38, *P* < 0.005, respectively), significant interval effects (*F*_1_,_6_ = 19.35, *P* < 0.0001 and *F*_1_,_6_ = 27.02, *P* < 0.0001, respectively), but not press-order x interval interactions. That is, the second-press speeds were significantly lower than the first-press speeds, and intervals reduced response speeds. **P* < 0.005, significantly slower than the 0-, 1- and 3-s interval values; ^$^*P* < 0.005, significantly slower than respective first lever-press value; %*P* < 0.005, significantly slower than first lever-press value.

**(B)** Mean percent of CR-schedule values. The 16-pulse data are described in Fig. 6C, which is also shown here in the left for easy comparison with the 8- and 4-pulse train data (middle and right panels, respectively). While variable x interval interactions were not significant, significant variable effects were present for 8- and 4-pulse trains (*F*_2_,_12_ = 32.79, *P* < 0.0001 and *F*_2_,_12_ = 11.05, *P* < 0.005, respectively). In all cases, first lever-press speeds were less affected by intervals than lever-press and reinforcement rates in a statistically significant manner (*P* < 0.001 and *P* < 0.0005, respectively for 8 pulse; *P* < 0.05 and *P* < 0.005, respectively for 4 pulse). Thus, these results confirm that first lever-press speeds after reinforcement incidents are less vulnerable to interval schedules than lever-press or reinforcement rates, and therefore the results suggest that photo-pulse trains exciting DA neurons briefly potentiate the initiation of conditioned response.

Supplementary Figure 2


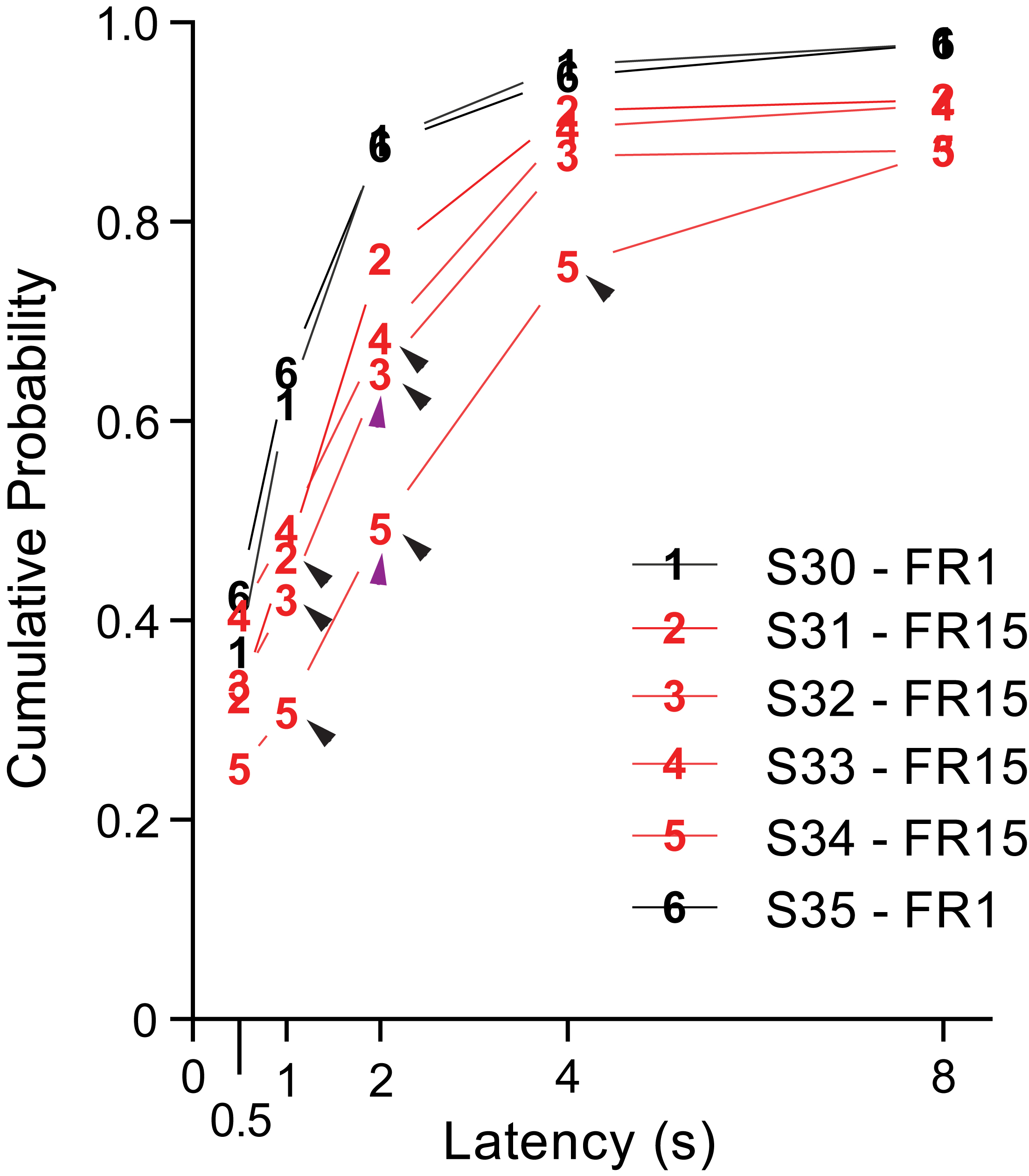


**Mean cumulative probabilities of the first lever-press after reinforcement incident over sessions 30-35**

To determine whether repeated experience with FR schedules attenuated the invigorating effect of phasic DA signals on conditioned approach response, we analyzed cumulative probabilities of the occurrence of first lever-presses after reinforcement incidents during sessions 30-35, with a 5_period_ x 6_session_ ANOVA. The results support the notion that the brief invigorating effect of photo-pulse trains on conditioned response decreased more and more with repeated sessions on the FR15 schedule. While first lever-press occurrences between the two FR1 sessions 30 and 35 did not differ from each other at respective time periods, some of their periods significantly differed from those of the FR15 sessions 31-34 (Tukey’s test after a significant period by session interaction, *F*_20_,_100_ = 2.46, *P* < 0.005). Specifically, the first FR15 session 31 reduced occurrence probability, compared to the FR1 session 35, at the 1-s period. The 2^nd^ FR15 session 32 reduced occurrence probabilities at the 1- and 2-s periods compared to both FR1 sessions 30 and 35. The 3^rd^ FR15 session 33 reduced occurrence probability at 2-s period compared to both sessions 30 and 36. Finally, the 4^th^ FR15 session 34 reduced occurrence probabilities at the 1-, 2-, and 4-s periods compared to sessions 30 and 35. Moreover, the 4^th^ FR15 session 34 reduced probabilities at the 2-s period compared to the 1^st^ or 3^rd^ FR15 sessions 31 and 33. Thus, these results support the notion that the brief potentiating effects of phasic excitation of DA neurons on the initiation of conditioned response diminish with repeated testing with the FR15 schedule. In other words, potentiating effect of photo-pulse trains on the initiation of conditioned response is suppressed as animals learn that the first response is never reinforced. Black arrowhead: *P* < 0.05, significantly lower than respective FR1 value; purple arrowhead: *P* < 0.05, significantly greater than respective session 31 value.
